# Contention-Less Multi-Link Synchronous Transmission for Throughput Enhancement and Heterogeneous Fairness in Wi-Fi 7

**DOI:** 10.3390/s24113642

**Published:** 2024-06-04

**Authors:** Lam Kwon, Eun-Chan Park

**Affiliations:** Department of Information and Communication Engineering, Dongguk University-Seoul, Seoul 04620, Republic of Korea; lamk@dongguk.edu

**Keywords:** multi-link operation, synchronous transmission, Wi-Fi 7, IEEE 802.11be

## Abstract

Multi-link operation (MLO) is a new and essential mechanism of IEEE 802.11be Extremely High Throughput (Wi-Fi 7) that can increase throughput and decrease latency in Wireless Local Area Networks (WLANs). The MLO enables a Multi-Link Device (MLD) to perform Simultaneous Transmission and Reception (STR) in different frequency bands. However, not all MLDs can support STR due to cross-link or in-device coexistence interference, while an STR-unable MLD (NSTR-MLD) can transmit multiple frames simultaneously in more than two links. This study focuses on the problems when NSTR-MLDs share a link with Single-Link Devices (SLDs). We propose a Contention-Less Synchronous Transmission (CLST) mechanism to improve fairness between NSTR-MLDs and SLDs while increasing the total network throughput. The proposed mechanism classifies links as MLD Dominant Links (MDLs) and Heterogeneous Coexistence Links (HCLs). In the proposed mechanism, an NSTR-MLD obtains a Synchronous Transmission Token (STT) through a virtual channel contention in the HCL but does not actually transmit a frame in the HCL, which is compensated for by a synchronous transmission triggered in the MDL. Moreover, the CLST mechanism allows additional subsequent transmissions up to the accumulated STT without further contention. Extensive simulation results confirm the outstanding performance of the CLST mechanism in terms of total throughput and fairness compared to existing synchronous transmission mechanisms.

## 1. Introduction

The ongoing next-generation Wireless Local Area Network (WLAN) standard, IEEE 802.11be Extremely High Throughput (EHT) [1], also called Wi-Fi 7, aims to achieve a maximum throughput of at least 30 Gbps. It has proposed various technologies to realize this goal. In the physical (PHY) layer, a bandwidth is extended up to 320 MHz, 4096 Quadrature Amplitude Modulation (QAM) is introduced, single-user Multiple Resource Unit (MRU) is adopted for flexible resource utilization, and preamble puncturing technology is proposed for channel efficiency. Various technologies have also been discussed in the Medium Access Control (MAC) layer, including multi-link operation (MLO), Multi-AP coordination, triggered Transmission Opportunity Sharing (TXS), restricted Target Wake Time (r-TWT), Hybrid Automatic Repeat Request (HARQ), and so on [2,3,4,5,6]. The IEEE 802.11be standard also aims to reduce worst-case latency and jitter for time-sensitive applications [7]. Among these technologies, MLO is one of the most representative mechanisms that can considerably increase throughput and/or decrease delay. This enhancement in throughput and delay will enable the fulfillment of the diverse requirements of sensor networks in dense environments such as smart cities or industrial Internet of Things (IoT). MLO allows devices to utilize multiple frequency bands (links). MLO defines two channel access modes: asynchronous mode and synchronous mode. The asynchronous mode allows a Multi-Link Device (MLD) to transmit frames independently on multiple links. It can also support Simultaneous Transmission and Reception (STR), allowing concurrent uplink and downlink transmissions. The STR operation can be constrained because cross-link interference or in-device coexistence interference cannot be effectively avoided due to hardware limitations or cost issues. The synchronous mode can be used for the STR-incapable MLD (referred to as NSTR-MLD). It can avoid cross-link or in-device interference problems by synchronizing the transmission durations in multiple links. On the other hand, a new unlicensed frequency band of 6 GHz (5.925–7.125 GHz) has been opened for WLAN devices since 2020. This wide bandwidth of 1200 MHz can be used by Wi-Fi 7 devices and Wi-Fi 6E devices, but the legacy single-link devices (SLDs) do not support this frequency band. Therefore, various heterogeneous Wi-Fi devices such as STR-MLD, NSTR-MLD, and SLD can share multiple links in various ways in 2.4 GHz, 5 GHz, and 6 GHz frequency bands.

This paper focuses on the problems faced by MLO’s synchronous transmission mechanisms when NSTR-MLDs share a link with SLDs. If the link is congested, SLDs may deprive NSTR-MLDs of synchronous transmission opportunities, preventing MLO’s benefits in terms of throughput enhancement from being fully exploited. At the same time, the channel access scheme of synchronous transmission can be biased toward SLDs or NSTR-MLDs. Thus, it is challenging to ensure fairness between NSTR-MLDs and SLDs. These problems are exacerbated as the level of contention becomes asymmetric between links. In this study, we assume saturated traffic scenarios and evaluate fairness using Jain’s fairness index, which is widely used in the literature [8].

To deal with these problems, we propose a *Contention-Less Synchronous Transmission* (CLST) mechanism for NSTR-MLD. Its objective is to improve the total throughput achieved by NSTR-MLDs and SLDs while attaining fairness between them in the shared link. We assume that legacy SLDs operate in the 2.4 GHz and 5 GHz frequency bands, while MLDs can employ any available frequency bands, including 6 GHz bands. Based on this assumption, we classify links as a *Heterogeneous Coexistence Links* (HCLs) and *MLD Dominant Links* (MDLs). The former are shared by NSTR-MLDs and many SLDs, whereas the latter has relatively few SLDs. The main idea of the CLST mechanism is to differentiate the transmission on the HCL and MDL such that the NSTR-MLD has more synchronous transmission chances with less contention and collision. For this purpose, we introduce a variable referred to as the *Synchronous Transmission Token* (STT). It plays a key role in improving total throughput and balancing fairness between NSTR-MLDs and SLDs. An NSTR-MLD acquires (increases) the STT when the transmission is blocked in the HCL and uses (decreases) it when the synchronous transmission is initiated from the MDL. In this manner, the CLST mechanism allows synchronous transmission in the HCL while reducing contention. Moreover, the CLST mechanism can improve fairness between NSTR-MLDs and SLDs by controlling the transmission in the HCL through STT. We performed extensive simulations and compared the performance of the proposed CLST mechanism with existing synchronous transmission mechanisms. It was confirmed that the proposed CLST mechanism improves the total throughput while improving fairness between NSTR-MLDs and SLDs. The contributions of this study are summarized as follows:

- With the novel concept of STT, the proposed mechanism can alleviate channel contention for synchronous transmission, time, and it can balance the transmission of NSTR-MLDs and SLDs in HCL.
- The proposed mechanism is flexible. It can be configured to enforce fairness further or improve throughput by adjusting the increment parameter of STT.
- Unlike the existing approach of synchronous transmission mechanisms, it does not modify the channel contention (i.e., backoff) procedure. It can be easily implemented without significant signaling overheads or complex computations.

The subsequent sections of this paper are organized as follows: Section 2 overviews the MLO defined in IEEE 802.11be and addresses its problems. The proposed CLST mechanism is presented in Section 3. In Section 4, the performance of the proposed mechanism is evaluated and compared to existing mechanisms through simulations. Finally, Section 5 concludes the paper.

## 2. Background and Problem Statement

### 2.1. Multi-Link Operation

The MLO is a new mechanism introduced in IEEE 802.11be that enables the utilization of a wide frequency spectrum. The MAC layer has been divided into the lower MAC (L-MAC) and upper MAC (U-MAC) sub-layers to support the MLO. The independent L-MAC handles each interface and supports link-specific operations, e.g., channel access and link adaptation. Meanwhile, a common U-MAC supports traffic-to-link allocation, frame aggregation or fragmentation, and sequence numbering. The MLO establishes independent PHY and L-MAC instances to formulate an interface that accommodates diverse frequency bands, and it can be implemented by reusing PHY and L-MAC layer functions and maintaining identical upper layers as the legacy 802.11 architecture [9]. Unlike the legacy system, where all bands cannot be used if the primary frequency band is occupied, each band can be accessed independently, achieving low latency, high throughput, and high reliability [10,11,12]. When using MLO, traffic needs to be distributed to different links by a traffic-to-link allocation policy, which is beyond the scope of this study.

The 802.11be has designed MLO to support STR; however, not all MLDs can support STR because of limitations in device size and implementation cost. We consider three heterogeneous devices as follows.

- STR-MLD: This device can operate multiple links independently, thus, full-duplex communication can be supported, as well as asynchronous transmission. Proper interference cancellation technology is essential in this type to avoid in-device coexistence interference. In IEEE 802.11be, the support of STR is mandatory for the Access Point (AP) but not for the non-AP (station).
- NSTR-MLD: This device does not support the STR capability; an ongoing transmission in one link prevents other links from receiving data. Asynchronous transmission is not desirable for this device, and synchronous transmission mechanisms have been proposed so that this device can transmit multiple frames in more than two links simultaneously.
- SLD: This is a legacy device that can use only one link.

In the literature, many studies have been conducted regarding MLO. The experimental studies in [13,14,15,16,17] have shown that the MLO effectively decreases the delay of MLD in diverse environments. It has been reported in [13,14,15] that the performance of MLO can be debased due to cross-link interference, high traffic load, severe contention, or coexistence with SLDs. In addition, the studies in [16,18] have shown that the delay gain of MLO diminishes as the number of links increases. Unlike the aforementioned studies, the studies in [19,20,21,22,23,24] have investigated the effect of MLO on the throughput enhancement. These studies mostly assume conditions of saturated traffic. The analytical model has been developed, and the theoretical throughput of NSTR-MLD has been derived when they coexist with SLDs [19,20]. Also, the study in [19] has evaluated the performance of several synchronous transmission mechanisms. The experimental study in [21] has confirmed that the performance of NSTR-MLD is degraded as the link is congested with many legacy SLDs, suggesting the need for advanced synchronous transmission mechanisms. In [22], a new channel access mechanism, AP-assisted synchronous transmission, has been proposed to improve the utilization of MLO. It makes use of Multi-User Request-To-Send (MU RTS) transmission opportunity sharing so that the AP can reserve the link for the synchronous transmission. The authors of [23,24] have proposed several schemes that give a penalty or compensation to backoff procedures for improving coexistence performance, and they have designed a new single-value metric for considering both throughput and latency gains. Moreover, the backoff overflow problem has been identified, and possible solutions to this problem have been proposed [24]. Our work is motivated by these previous studies [19,20,21,22,23,24], and it is different from them in that the proposed mechanism does not modify the standard backoff procedure. The approach to controlling the backoff procedure is prone to bias against MLDs or SLDs, and it is difficult to strike a balance between them in diverse environments. Instead, the proposed mechanism introduces the novel idea of STT, which is obtained after the virtual contention in the HCL and consumed on multiple synchronous transmissions triggered after the contention in the MDL. In this process, the average backoff time can decrease, improving throughput and delay.

### 2.2. Blocking Issue in NSTR-MLD

In NSTR-MLD, an ongoing transmission in one link causes power leakage, making the other link unavailable for receiving frames due to in-device interference. It is also unavailable for transmitting frames because it is determined to be busy. We define this problem as the *blocking* of NSTR-MLD.

**Figure 1:**
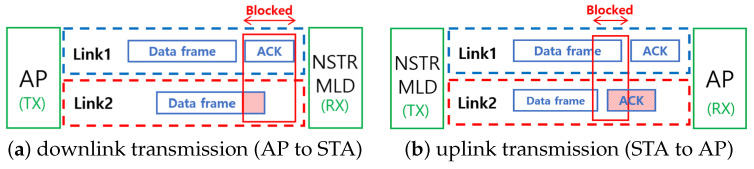
Illustration of the blocking problem in NSTR-MLD.

Figure 1 illustrates the problem related to blocking. In the case of a downlink transmission from AP to station (STA) in Figure 1a, the blocking results from the transmission of ACK in Link1 corrupt the data frames that are being received in Link2. This problem happens in a similar way in the case of uplink transmission, as shown in Figure 1b. It is possible that the STA can transmit frames at the same time in two links; however, the transmission in Link1 probably leads to the failure of ACK reception in Link2. Even if the transmission of Link2 is successful, it is considered failed. This ACK failure causes unnecessary retransmission and increases the Contention Window (CW) according to the Binary Exponential Backoff (BEB) mechanism. We performed preliminary simulations to observe the performance of NSTR-MLD and verified the problems of asynchronous transmission of NSTR-MLD; i.e., (i) even in collision-free and SLD-free ideal cases, NSTR-MLD achieves about only 30% higher throughput than SLD, and (ii) if a link is shared with many SLDs, SLD rather obtains up to two times higher throughput than NSTR-MLD. These results confirm that the asynchronous MLO cannot fully obtain the gain of MLO due to blocking.

### 2.3. Synchronous Transmission Mechanisms

**Figure 2:**
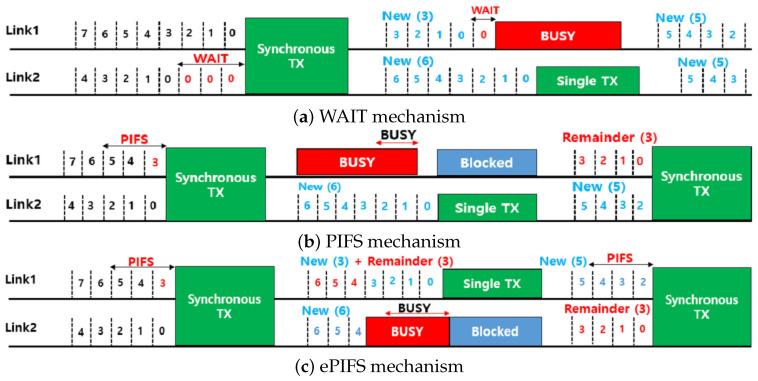
Demonstration of existing synchronous transmission mechanisms.

To handle the blocking problem of asynchronous MLO, the IEEE 802.11be discussed three synchronous transmission mechanisms, which are named WAIT, PIFS, and ePIFS, and details are described below [25,26,27]. Note that they were discussed during the standardization process, and it is expected that the WAIT mechanism would be included in the final standard. Figure 2 illustrates examples of three synchronous transmission mechanisms’ operations.

- WAIT (Figure 2a): If a contention finishes in Link2 but the contention is ongoing in Link1, the transmission is delayed, and a synchronous transmission starts when the contention (i.e., backoff procedure) is completed in both links. If Link1 is detected as busy while waiting for the completion of the contention in Link2, a new backoff procedure starts in Link1 after it becomes idle.
- PIFS (Figure 2b): When the backoff procedure in Link2 is terminated, a synchronous transmission is attempted in both links if Link1 is idle for the Point Coordination Function Interframe Space (PIFS) time. Even though the contention has not yet ended in Link1, the transmission in Link1 is allowed, called *free-riding*, and the remaining backoff counter is maintained and used in the next channel contention.
- ePIFS (Figure 2c): This mechanism is slightly different from PIFS in that the penalty is given to the free-riding transmission. As shown in Figure 2c, after the free-riding transmission in Link1, the remainder of the backoff counter is added to a new backoff counter in the next transmission.

We discuss and compare three synchronous mechanisms in terms of fairness and throughput. In the WAIT mechanism, the synchronous transmission can start after contention for the link with the largest backoff counter value. Therefore, the backoff time of MLD probably would be larger than that of SLD. When MLDs share the link with SLDs, the transmission opportunities can be biased toward SLDs. In addition to this problem, the WAIT mechanism has a weakness in terms of throughput when the contention level is asymmetric in the links. Due to the BEB mechanism, frequent collisions in Link1 increase the CW size. Then, the MLDs employing both links have to wait a long time before synchronous transmission, which decreases the efficiency of Link2. Therefore, the MLDs employing both links have to wait a long time before synchronous transmission, which decreases the efficiency of Link2.

In contrast to the WAIT mechanism, the PIFS mechanism allows synchronous transmission based on the smallest backoff counter. Thus, MLD may have more transmission opportunities than SLD. The unfairness problem arises in the opposite aspect of the WAIT mechanism. Note that the BEB mechanism cannot effectively deal with the collisions in Link1 because even though the CW of devices in Link1 increases, the free-riding transmission of MLD is initiated by the contention in Link2 instead of Link1.

The ePIFS mechanism is proposed to alleviate this problem of the PIFS mechanism. However, the problem cannot be effectively resolved. The free-riding transmission of MLD in a more-contending link (e.g., Link1) would probably be triggered by the termination of the backoff procedure in a less-contending link (e.g., Link2). Even though the ePIFS mechanism gives a penalty to MLD in Link1, this penalty is not effective in Link2. Consequently, MLDs still probably have more transmission chances than SLDs [24].

## 3. Contention-Less Synchronous Transmission Mechanism

We propose the contention-less synchronous transmission mechanism to effectively deal with the problems of NSTR-MLDs when they coexist with SLDs. Its objective is two-fold: (i) increasing the total throughput for NSTR-MLDs and SLDs and (ii) improving fairness between them. The first objective can be achieved by increasing the chances of synchronous transmissions while making the synchronous transmissions performed in a contention-less manner. The second one can be carried out by maintaining an independent backoff procedure for each link and by controlling free-riding transmission due to synchronous transmission. The proposed mechanism is designed to achieve these two objectives in a unified framework by introducing a new variable called *STT*. It is assumed that SLDs usually operate in the HCL, but the MDL has few SLDs, so the contention of HCL is relatively higher than MDL.

### 3.1. Improving Fairness between NSTR-MLD and SLD

**Figure 3:**
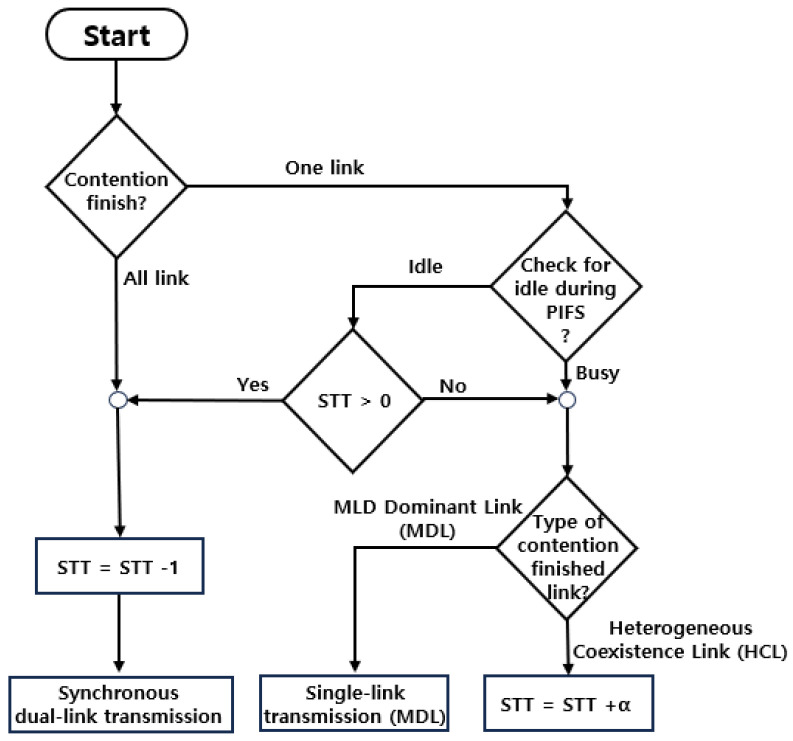
Flowchart of the proposed CLST mechanism.

Figure 3 shows the flow chart of the CLST mechanism. Each NSTR-MLD maintains STT. Its initial value is zero. If the NSTR-MLD finishes the backoff procedure in the HCL but the MDL is not idle during the PIFS time, the value of STT is increased by a predetermined value of α without transmitting a frame in the HCL. On the other hand, when the synchronous transmission is triggered from the MDL (i.e., the backoff procedure is completed in the MDL and the HCL is idle during the PIFS time), and if the value of STT is positive, the synchronous transmission is performed, and the value of STT is decreased by one. As long as the value of STT is zero or negative, the NSTR-MLD is not allowed to transmit a data frame in the HCL. However, regardless of the value of STT, the NSTR-MLD can transmit a frame in the MDL. In summary, unlike PIFS and ePIFS mechanisms, the CLST mechanism does not unconditionally allow free-riding transmission because synchronous transmission is not permitted if there are no valid STTs. Therefore, the CLST mechanism can improve the fairness between NSTR-MLD and SLD in HCL.

### 3.2. Improving Multi-Link Throughput

In the proposed CLST mechanism, even though the backoff procedure is finished in the HCL, the transmission in the HCL is blocked, but the STT is increased by α if MDL is not idle during the PIFS time. We design the CLST mechanism so that the blocking of transmission in the HCL can be compensated for through the synchronous transmission triggered by the MDL.

For this purpose, we propose an *Extra Compensation Transmission* (ECT) scheme, which can be simply incorporated into the framework of the CLST mechanism. The ECT scheme works as follows. When the NSTR-MLD finishes the contention and transmission in the MDL, the NSTR-MLD checks whether the link is idle during the PIFS time. If it is determined to be idle, the synchronous multi-link transmissions are repeated without additional contentions. Since the PIFS time (25 μs) is shorter than the DIFS time (34 μs), the extra transmission is not interfered with by a data frame transmission by other devices, whose backoff procedure can start after the DIFS time. Here, the next repeated transmission is not permitted if the previous transmission fails. The maximum number of repeated transmissions is limited to the fixed value of ECT. In this process, the value of STT is decreased by one for each transmission in the HCL. If there is no available STT (i.e., STT ≤ 0), the transmission in the HCL stops. Note that the extra transmissions are performed in the MDL up to ECT, regardless of STT, under the rationale that the contention is not severe and there are few SLDs in the MDL.

**Figure 4:**
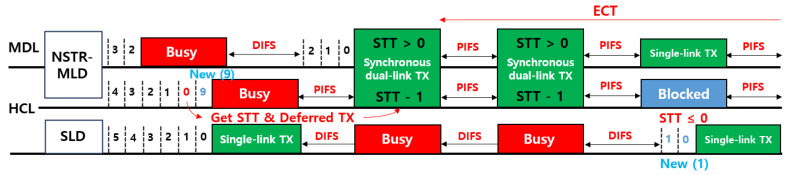
An operation example of the CLST mechanism.

Figure 4 shows a representative operation example of the CLST mechanism. After the contention is finished in the HCL but the MDL is busy, the transmission in the HCL is not allowed, but the STT is increased. The blocked transmission in the HCL is later triggered and compensated for by the contention in the MDL. When the contention is finished in the MDL, and the HCL is idle during the PIFS time, the synchronous multi-link transmission is performed, and subsequent multi-link transmissions are followed up to ECT in the MDL and up to the remaining STT in the HCL. We can summarize the advantages of the CLST mechanism employing STT and ECT as follows.

- The transmission in the HCL is triggered by the contention in the MDL, which effectively decreases the backoff time because the MDL usually has a lower contention level than the HCL.
- The add-on ECT scheme allows multiple synchronous transmissions in both HCL and MDL without further contention, contributing to the increase in throughput.
- The STT is earned due to the blocking in the HCL and consumed by the transmission in the HCL. Its value is not affected by the transmission in the MDL. Therefore, the CSLT mechanism can control fairness between NSTR-MLD and SLD in the HCL while improving the utilization of MDL.

### 3.3. Discussions of the CLST Mechanism

The key point of the CLST mechanism is to determine the values of α and ECT. Note that the value of α does not necessarily have to be a natural number; it can be any positive real number. If α is large and greater than one, the NSTR-MLD can have more synchronous transmission opportunities, contributing to the increase in total throughput. Conversely, as α becomes smaller, the transmission of NSTR-MLD in the HCL is blocked more, and SLDs can perform more transmissions. As the number of NSTR-MLDs ($N_{MLD}$) increases, the contention becomes severe, and the probability that each NSTR-MLD finishes the contention in the MDL decreases the chance of transmission. If the number of SLDs ($N_{SLD}$) is equal to $N_{MLD}$, we consider that it is desirable to set α to one. Based on these intuitions, we can conclude that α needs to be proportional to $N_{MLD}$ and inversely proportional to $N_{SLD}$. We simply set α to $N_{MLD}/N_{SLD}$. To implement this, the AP needs to determine α and broadcast it through a control frame. On the other hand, the ECT limits the maximum number of additional transmissions. In setting the ECT value, we can expect a trade-off between throughput and fairness. A large ECT can increase total throughput at the cost of fairness between NSTR-MLDs and SLDs in the HCL. Increasing ECT causes the burst transmission of data frames, which increases jitter and is not desirable with respect to real-time traffic. The effects of α and ECT are investigated in Section 4.2. Deriving their optimal values or devising dynamic algorithms to control them could be carried out in our future work.

We expect that the CLST mechanism can be straightforwardly extended to the case where the number of links is greater than two. In this case, the one link with the least SLDs is classified as MDL, and the remaining links are classified as HCL. We can consider two policies for managing the STT: (i) a common STT for all the HCLs and (ii) an individual STT for each HCL. With the first policy, a common STT is increased or decreased when the transmission in any HCL is blocked or performed. On the other hand, with the second policy, the individual STT is managed based on the blocking and transmitting in the corresponding link. The first policy is effective in maximizing the multi-link throughput, whereas the second one is desirable in maintaining per-device fairness on each link. Similar to the case of two links, the multi-link transmission involving more than one HCL is initiated by the contention in the MDL. Here, the traffic-to-link allocation mechanism becomes important to maximize the multi-link throughput or enforce per-device fairness, which could be carried out in our future work.

## 4. Simulation Results

In this section, we evaluate the performance of the proposed CLST mechanism by conducting extensive simulations. First, we compare the performance of the CLST mechanism with the existing synchronous transmission mechanisms in Section 4.1, and then, in Section 4.2, we investigate the effect of key parameters in the CLST mechanism on its performance.

Table 1 lists the configurations and parameters used in the simulation, considering the IEEE 802.11ax standard. In order to focus on the performance MLO, the simulation was performed under the following assumptions: (i) the channel is ideal, i.e., the transmission fails only due to collisions; (ii) the frame size and transmission rate are fixed; and (iii) all the devices always have data frames to transmit. We also considered that only MLDs exist in Link1 operating in the 6 GHz band, whereas MLDs and SLDs share Link2 operating in the 5 GHz band. All the MLDs were assumed to be NSTR-MLDs. Considering the design rationale of parameters of the CLST mechanism in Section 3.3, α was set to $N_{MLD}/N_{SLD}$, and ECT was set to 6.

We implemented the simulator with MATLAB by considering key features of MLO and IEEE 802.11 evaluation methodology documents. We established several performance indices for evaluating throughput and fairness as follows.

- $th_{MLD}$ and $th_{SLD}$: The average per-device throughput achieved by NSTR-MLDs and SLDs in Link2, respectively.
- $TH_{MLD}$ and $TH_{SLD}$: The total throughput achieved by NSTR-MLDs and SLDs in Link2, respectively.
- $TH_{L2}$: The total throughput achieved by all the devices in Link2 where NSTR-MLDs and SLD coexist.
- $TH_{T}$: The network-wide total throughput is achieved by all the devices in all the links.
- *F*: Jain’s fairness index, i.e.,

$$F=\frac{{\left(\sum_{i=1}^{N}th_{i}\right)}^{2}}{N\sum_{i=1}^{N}th_{i}^{2}},$$

where *N* is the total number of devices in Link2 and $th_{i}$ is the throughput achieved by device *i* in Link2.

### 4.1. Performance Comparison of the CLST Mechanism

We focused on the performances and problems of the existing synchronous transmission mechanisms and compared them with the proposed CLST mechanism in terms of fairness and throughput.

#### 4.1.1. Performance Evaluation in Terms of Fairness

**Figure 5:**
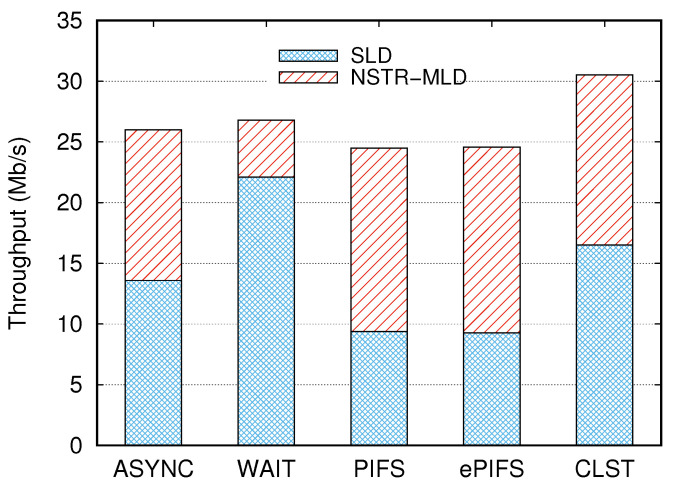
Comparison of throughputs achieved by NSTR-MLDs and SLDs in Link2 for several synchronous transmission mechanisms.

In the first simulation, we set $N_{MLD}$ and $N_{SLD}$ to 15 so that the same number of MLDs and SLDs share Link2, whereas there wareere no SLDs in Link1, and we compared $TH_{MLD}$ and $TH_{SLD}$ to evaluate the fairness between NSTR-MLDs and SLDs. Figure 5 shows a stacked histogram consisting of $TH_{MLD}$ and $TH_{SLD}$. The difference between $TH_{MLD}$ and $TH_{SLD}$ was the smallest at 1.18 Mb/s for the asynchronous transmission mechanism (denoted as ASYNC) and the largest at 17.42 Mb/s for the WAIT mechanism. The unfairness problem between MLDs and SLDs occurred in the WAIT, PIFS, and ePIFS mechanisms that operate based on the backoff counter value of one link. The throughput was mostly achieved by SLDs in the WAIT mechanism. However, MLDs achieved higher throughput than SLDs in the PIFS and ePIFS mechanisms. The CLST mechanism reduced the difference between $TH_{MLD}$ and $TH_{SLD}$ to 2.51 Mb/s while increasing the total throughput by 17% compared to the ASYNC mechanism.

**Figure 6:**
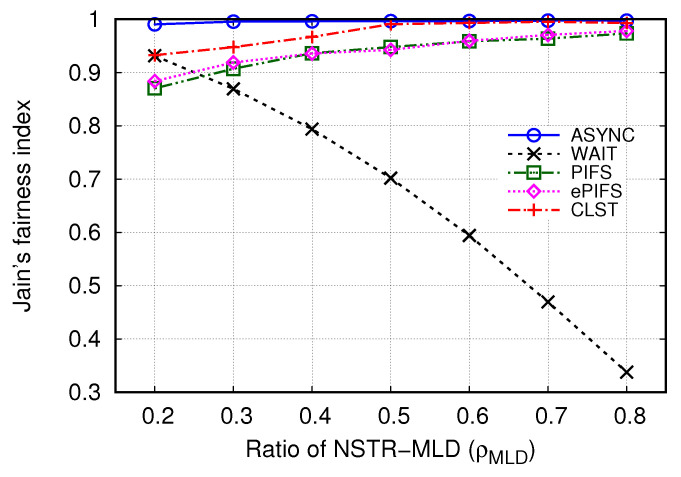
Comparison of Jain’s fairness index for several synchronous transmission mechanisms.

We further investigated the fairness between NSTR-MLDs and SLDs in a wider range of $\rho_{MLD}$. Here, we fixed the number of total devices (NSTR-MLD and SLD) in Link2 to 30 and changed the number of NSTR-MLDs from 6 to 24 so that $\rho_{MLD}$ would range from 0.2 to 0.8. Note that as $\rho_{MLD}$ has a smaller value, the degree of link contention becomes more asymmetric. For example, if ρ = 0.2, Link1 has 6 NSTR-MLDs, and Link2 has 6 NSTR-MLDs and 24 SLDs. Figure 6 shows Jain’s fairness index (*F*) when $\rho_{MLD}$ is between 0.2 and 0.8. In terms of *F*, the asynchronous transmission mechanism outperformed the other mechanisms; its value was at least 0.99 for the entire range of $\rho_{MLD}$. However, in the case of the WAIT mechanism, the unfairness problem was severe, especially as $\rho_{MLD}$ increased; i.e., *F* decreased from 0.93 to 0.34 when $\rho_{MLD}$ increased from 0.2 to 0.8. Unlike the WAIT mechanism, the PIFS and ePIFS mechanisms showed that the unfairness problem was exacerbated as $\rho_{MLD}$ decreased. When $\rho_{MLD}$ = 0.2, the *F* values of PIFS and ePIFS were 0.87 and 0.88, but they increased to 0.97 and 0.98, respectively. The CLST mechanism alleviated the unfairness problem so that *F* ranged between 0.97 and 0.99 when $\rho_{MLD}\geq$ 0.4. Although *F* was somewhat smaller than one when $\rho_{MLD}$ was small, we expect that the CLST mechanism can further improve fairness by setting the increment of STT (α) to a smaller value.

#### 4.1.2. Performance Evaluation in Terms of Efficiency

**Figure 7:**
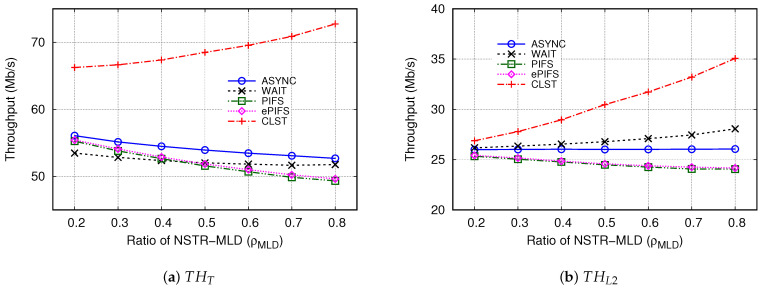

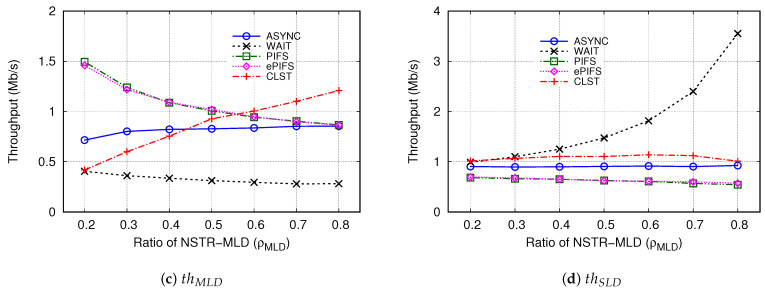
Comparison of throughputs for several synchronous transmission mechanisms.

In order to evaluate the performance in terms of multi-link efficiency, we observed various performance indices, i.e., $TH_{T}$, $TH_{L2}$, $th_{MLD}$, and $th_{SLD}$, which arre shown in Figure 7 when $\rho_{MLD}$ changed between 0.2 and 0.8.

First, we observe the total network-wide throughput that is obtained by all the devices in Link1 and Link2 ($TH_{T}$), which is shown in Figure 7a. The CLST mechanism showed outstanding performance in terms of $TH_{T}$ for the entire range of $\rho_{MLD}$. As $\rho_{MLD}$ increased from 0.2 to 0.8, the $TH_{T}$ of the CLST mechanism increased from 66 Mb/s to 73 Mb/s, whereas the $TH_{T}$s of the other existing mechanisms of $TH_{T}$ gradually decreased with respect to the increase in $\rho_{MLD}$. The reason is as follows. The increase in $\rho_{MLD}$ leads to frequent collisions in Link1, thus reducing the throughput achieved in Link1. However, in the case of CLST, the increase in $\rho_{MLD}$ can mitigate collisions in Link2 and increase its throughput because NSRT-MLDs are not directly involved in the channel contention in Link2, which can be confirmed in Figure 7b Compared to the ASYNC and ePIFS mechanisms, the CLST mechanism increased $TH_{T}$ by about 18–38% and by about 20–47%, respectively.

Figure 7b shows that when $\rho_{MLD}$ = 0.2, there was no significant difference in $TH_{L2}$ across all the mechanisms. However, as $\rho_{MLD}$ increased, the $TH_{L2}$ of each mechanism changed in a different way. In the ASYNC mechanism, $TH_{L2}$ was hardly affected by the change in $\rho_{MLD}$ and maintained an almost constant value of 26 Mb/s. As $\rho_{MLD}$ increased from 0.2 to 0.8, the $TH_{L2}$ of the WAIT mechanism increased and was higher than that of the ASYNC mechanism, up to 8% when $\rho_{MLD}$ = 0.8. In the cases of PIFS and ePIFS mechanisms, the $TH_{L2}$ of ePIFS was slightly higher than that of PIFS, but the difference was negligible, and it decreased from 25.4 Mb/s to 24.2 Mb/s as $\rho_{MLD}$ increased from 0.2 to 0.8 and was lower than that of the ASYNC mechanism by about 6–7% when $\rho_{MLD}\geq$ 0.5. On the other hand, as $\rho_{MLD}$ increased, the $TH_{L2}$ of the CLST mechanism almost linearly increased from 26.9 Mb/s to 35.1 Mb/s. Compared to the ASYNC, WAIT, and ePIFS mechanisms, the CLST mechanism increased the $TH_{L2}$ up to 35%, 25%, and 45%, respectively.

In addition to the total throughput in terms of $TH_{T}$ and $TH_{L2}$, we observed the average per-device throughput of NSTR-MLDs and SLDs, $th_{MLD}$, and $th_{SLD}$. As shown in Figure 7c,d, in the CLST mechanism, the $th_{MLD}$ increased from 0.42 Mb/s to 1.21 Mb/s with respect to the increase in $\rho_{MLD}$, while $th_{SLD}$ did not change much regardless of $\rho_{MLD}$ and was almost 1.1 Mb/s. These results imply that, in the CLST mechanism, the increase in NMLD improves the possibility of synchronous transmission without exacerbating collisions in Link2. Therefore, as long as the number of total devices does not change, each SLD maintains almost constant throughput in the CLST mechanism. In the ASYNC mechanism, both $th_{MLD}$ and $th_{SLD}$ were not much affected by the change of $\rho_{MLD}$. However, in the WAIT mechanism, $th_{MLD}$ had the least value for the whole range of $\rho_{MLD}$, and it slightly decreased as $\rho_{MLD}$ increased. Inversely, the WAIT mechanism had the largest value of $th_{SLD}$ among all the mechanisms, and it sharply increased from 0.99 Mb/s to 3.55 Mb/s as $\rho_{MLD}$ increased. These results reconfirm the significant unfairness between MLDs and SLDs in the WAIT mechanism. Unlike the WAIT mechanism, in the ePIFS mechanism, $th_{MLD}$ greatly decreased from 1.46 Mb/s to 0.86 Mb/s, but $th_{SLD}$ slightly decreased from 0.69 Mb/s to 0.57 Mb/s.

### 4.2. Effects of Key Parameters of the CLST Mechanism

#### 4.2.1. Effect of STT Increment (α)

It is worth noting that in the CLST mechanism, the increase in α leads to aggressive multi-link transmission, which contributes to the increase in $th_{MLD}$ and $TH_{L2}$, but it may worsen the fairness between MLDs and SLDs. Here, we investigate the effect of α on the throughput and fairness of the multi-link operation.

**Figure 8:**
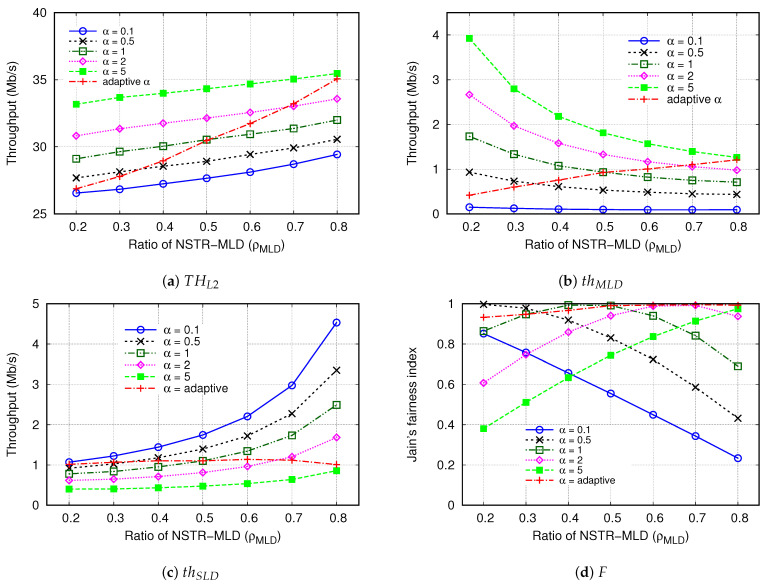
Effect of STT increment (α) on throughput and fairness.

Figure 8 compares $TH_{L2}$, $th_{MLD}$, $th_{SLD}$, and *F* for various fixed values of α. Recall that, in the previous simulations, we set α to $N_{MLD}/N_{SLD}$ so that it changes depending on $\rho_{MLD}$, and this case is also included in Figure 8 and denoted as *adaptive α*. In this simulation, the value of ECT was set to 6, the same as in the previous simulations. When α was large, NSTR-MLDs were able to acquire STT quickly and increased the synchronous transmission opportunities, increasing $TH_{L2}$ accordingly. Moreover, $TH_{L2}$ almost linearly increased with $\rho_{MLD}$, and the slope of $TH_{L2}$ was similar in all the cases of fixed α. However, the values of $TH_{L2}$ with adaptive α were comparable to those with α = 0.1 and 5 when $\rho_{MLD}$ = 0.2 and 0.8, respectively.

In a similar way, $th_{MLD}$ and $th_{SLD}$ are shown in Figure 8b and Figure 8c, respectively. Except for the case of adaptive α, $th_{MLD}$ increased for a larger value of α. Meanwhile, when α = 0.1, $th_{MLD}$ changed little despite the increase in $\rho_{MLD}$. This result is due to the fact that the NSTR-MLD could not have sufficient STT when it had a transmission chance. Unlike the cases of fixed α, $th_{MLD}$ with adaptive α linearly increased from 0.42 Mb/s to 1.21 Mb/s when $\rho_{MLD}$ increased from 0.2 to 0.8. This is because, for adaptive α, the growth rate of STT in NSTR-MLD is proportional to $N_{MLD}$ so that NSTR-MLD can transmit more frames when they have a transmission opportunity.

Figure 8c shows that the results of $th_{SLD}$ were opposite to those of $th_{MLD}$. If α was smaller or $\rho_{MLD}$ increased, $th_{SLD}$ increased. The reason for these results can be explained as follows. As the number of NSTR-MLDs increases, the contention in MDL becomes severe. Even if an NSTR-MLD successfully finishes the contention in the HCL, it does not transmit a frame immediately; the transmission is delayed until the contention is completed in the MDL. However, an SLD is allowed to transmit a frame after the contention in the HCL. Therefore, due to the increase in $\rho_{MLD}$, $th_{MLD}$ decreases, but $th_{SLD}$ increases in the CLST mechanism. By controlling the value of α depending on the number of MLDs, in the case of adaptive α, $th_{SLD}$ values were not much affected by the change of $\rho_{MLD}$; it was between 1.01 Mb/s and 1.14 Mb/s for the whole range of $\rho_{MLD}$.

Figure 8d shows the effect of α on the fairness index. In the case of α = 0.1, *F* linearly decreased from 0.85 to 0.23 as $\rho_{MLD}$ increased from 0.2 to 0.8. Conversely, in the case of α = 5, *F* dropped below 0.5 when $\rho_{MLD}\leq$ 0.3, but it was larger than 0.9 when $\rho_{MLD}\geq$ 0.7. Thus, it is desirable to set α in proportion to $N_{MLD}$ to improve fairness. In the case of α = 1, *F* increased and was greater than 0.99 when $\rho_{MLD}$ = 0.4–0.5, meaning that setting α to one is appropriate when the number of MLDs is comparable to that of SLDs. If $\alpha =N_{MLD}/N_{SLD}$ (adaptive α), *F* was greater than 0.99 when $\rho_{MLD}\geq$ 0.5 and at least 0.93 for the entire range of $\rho_{MLD}$. The results in Figure 8d confirm that it is reasonable to set α as $N_{MLD}/N_{SLD}$ in terms of fairness.

**Figure 9:**
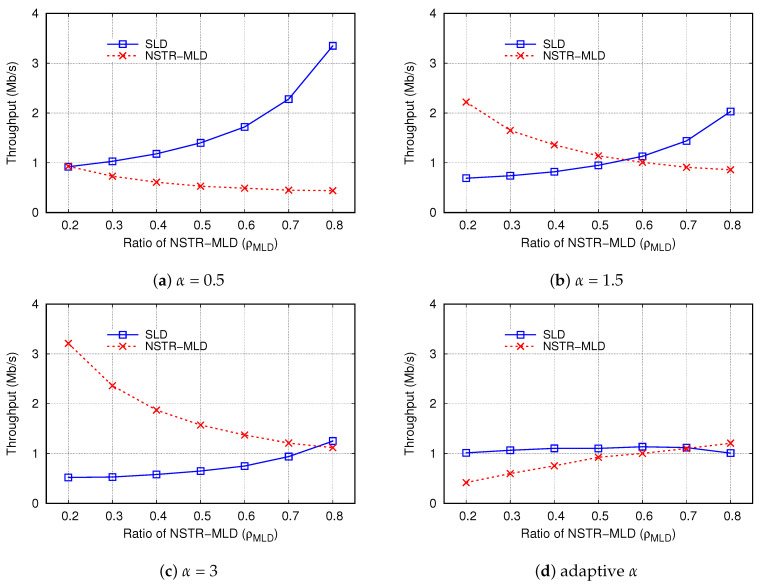
Comparison of the throughputs of NSTR-MLD and SLD for various values of α.

Figure 9 compares $th_{MLD}$ and $th_{SLD}$ for various values of α, including the case of adaptive α. When α was fixed (Figure 9a–c), $th_{MLD}$ decreased, but $th_{SLD}$ increased with respect to the increase in $\rho_{MLD}$ at the point where two graphs of $th_{MLD}$ and $th_{SLD}$ intersect, and NSTR-MLDs and SLDs have the same average throughput. We define this point as an *ideal fairness point*. From Figure 9a–c, we can see that the values of the horizontal axis ($\rho_{MLD}$) at the ideal fairness point were 0.20, 0.56, and 0.77 when α = 0.5, 1.5, and 3, respectively. This result implies that when the fraction of MLD among all the devices is high, the high value of α is effective in improving fairness between MLDs and SLDs. At this ideal fairness point, $th_{MLD}=th_{SLD}$ = 0.92, 1.06, and 1.15 Mb/s when α = 0.5, 1.5, and 3, respectively. On the other hand, in the case of adaptive α (Figure 9d), the difference between $th_{MLD}$ and $th_{SLD}$ greatly decreased, confirming that the adaptive setting of α can improve fairness. At the ideal fairness point, $th_{MLD}$ = $th_{SLD}$ = 1.11 Mb/s, which was not much different from the cases when α was fixed.

#### 4.2.2. Effect of ECT

Now, we analyze the effect of ECT on the performance of the CSLT mechanism. When the value of ECT is larger, an NSTR-MLD can transmit more frames once the transmission is possible after the contention in the MDL.

**Figure 10:**
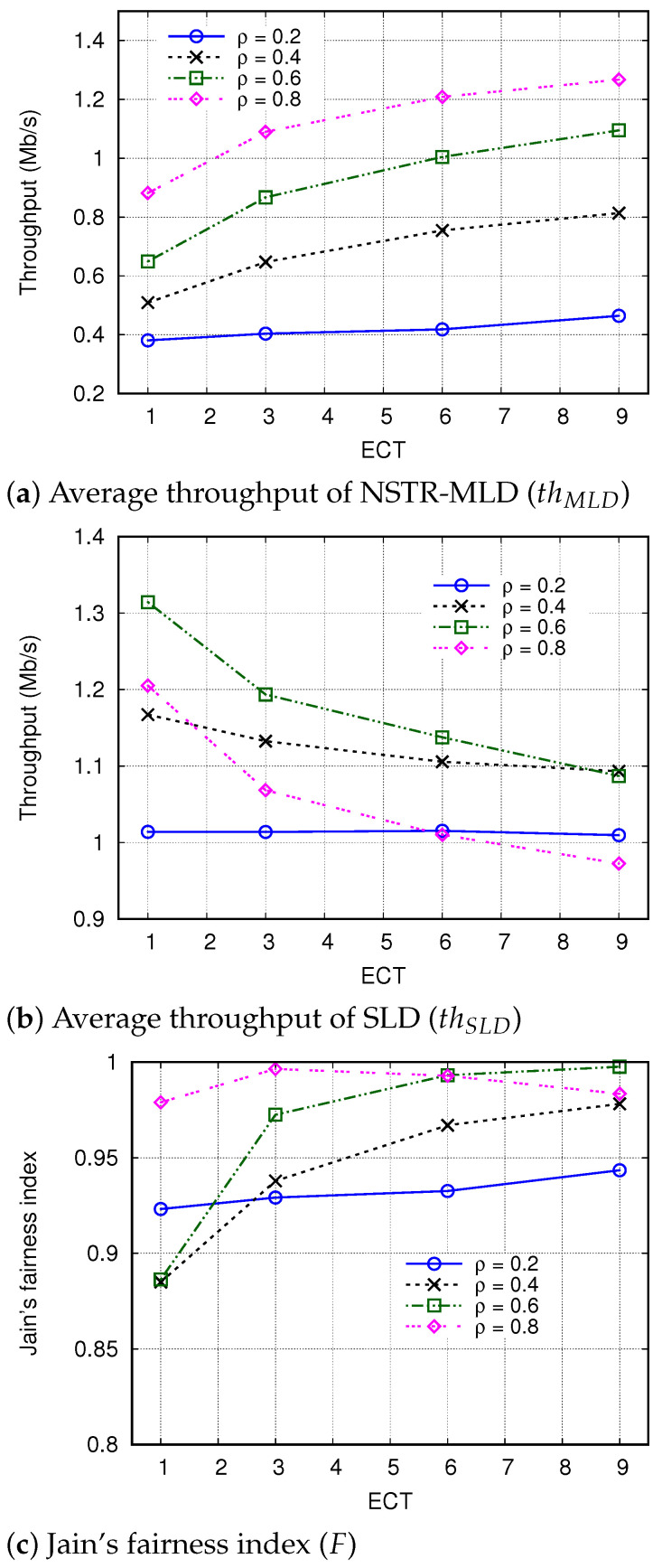
Effect of ECT on throughput and fairness.

Figure 10 shows $th_{MLD}$ and $th_{SLD}$ when the value of ECT increases from 1 to 9. The effect of ECT on $th_{MLD}$ and $th_{SLD}$ is not significant when $\rho_{MLD}$ is small; that is, even though ECT increased from 1 to 9, the maximum differences in the values of $th_{MLD}$ and $th_{SLD}$ were only 0.08 Mb/s and 0.01 Mb/s, respectively, when $\rho_{MLD}$ = 0.2. The effect of ECT became enlarged as $\rho_{MLD}$ increased. For example, when $\rho_{MLD}$ = 0.8, those differences in $th_{MLD}$ and $th_{SLD}$ were increased to 0.39 Mb/s and 0.23 Mb/s, respectively. By comparing Figure 10a,b, we can see that the increase in ECT leads to the increase in $th_{MLD}$ and the decrease in $th_{SLD}$. From these results, we can conclude that (i) when the number of MLDs is small, even though the ECT is large, the actual number of frames transmitted in the HCL is limited and controlled by the STT, and (ii) when the number of MLDs is large, the increase in ECT is effective in improving the throughput of NSTR-MLDs.

The effect of ECT on *F* can be observed in Figure 10c. When $\rho_{MLD}$ = 0.4–0.6, the increase in ECT contributes to improving fairness; e.g., when ECT increased from 3 to 9, *F* increased from 0.97 to 0.998 in the case of $\rho_{MLD}$ = 0.6. However, *F* was not much affected by ECT when $\rho_{MLD}$ = 0.2, and excessive increase in ECT rather impairs fairness when $\rho_{MLD}$ = 0.8; that is, *F* decreased from 0.997 to 0.984 when ECT increased from 3 to 9.

## 5. Conclusions

This study investigated the problems of MLO in IEEE 802.11be WLANs (Wi-Fi 7). When NSTR-MLDs and SLDs coexist in a link, the existing synchronous transmission mechanisms for NSTR-MLD cannot effectively utilize all the links and impair fairness between NSTR-MLDs and SLDs. In order to solve these problems, we proposed the CLST mechanism, whose key points can be summarized as follows.

- The links are classified as HCL and MDL, and the transmission is differentiated depending on the link type.
- By introducing a novel concept of STT, the transmission of NSTR-MLD in HCL can be controlled fairly and efficiently.
- The synchronous transmission in the HCL is initiated by contention in the MDL, and the contention of subsequent synchronous transmissions is mitigated by ECT.

Through the simulation study, we have confirmed that, compared to the existing mechanisms, the proposed mechanism increased the total network throughput by more than 40% while improving fairness between NSTR-MLDs and SLDs. This work can be extended to support various applications that require high throughput or low latency in sensor networks or IoT systems. In future work, we will devise advanced and dynamic algorithms to control STT and ECT, or we will find their optimal values by developing an analytical model. In addition, we plan to extend our study to reduce the delay of real-time traffic by developing an efficient traffic-to-link allocation mechanism and integrating it with the proposed CLST mechanism.

## Figures and Tables

**Table 1:** Simulation parameters.

Parameter	Value
Frequency band	6 GHz (Link1), 5 GHz (Link2)
Channel bandwidth	20 MHz
Frame size	1000 bytes
Transmission rate	98 Mb/s
slot time	9 μs
PIFS time	25 μs
Minimum contention window	7
Maximum contention window	1023
Number of devices in Link1	MLD (6–24)
Number of devices in Link2	MLD (6–24) and SLD (24–6)

## Data Availability

Data are contained within the article.

## Abbreviations

The following abbreviations are used in this manuscript:

AP	Access Point
ACK	Acknowledgement
BEB	Binary Exponential Backoff
CLST	Contention-Less Synchronous Transmission
CW	Contention Window
DIFS	Distributed Coordination Function InterFrame Space
ECT	Extra Compensation Transmission
EHT	Extremely High Throughput
HCL	Heterogeneous Coexistence Link
L-MAC	Lower-Medium Access Control
MAC	Medium Access Control
MDL	MLD Dominant Link
MLD	Multi-Link Device
MLO	Multi-Link Operation
PHY	Physical layer
PIFS	Point Coordination Function InterFrame Space
SLD	Single-Link Device
STA	STAtion
STR	Simultaneous Transmission and Reception
STT	Synchronous Transmission Token
TXOP	Transmission Opportunity
U-MAC	Upper-Medium Access Control
WLAN	Wireless Local Area Network
